# Animal Movement Prediction Based on Predictive Recurrent Neural Network

**DOI:** 10.3390/s19204411

**Published:** 2019-10-11

**Authors:** Jehyeok Rew, Sungwoo Park, Yongjang Cho, Seungwon Jung, Eenjun Hwang

**Affiliations:** School of Electrical Engineering, Korea University, 145, Anam-ro, Seongbuk-gu, Seoul 02841, Korea; rjh1026@korea.ac.kr (J.R.); psw5574@korea.ac.kr (S.P.); dydwkd486@korea.ac.kr (Y.C.); jsw161@korea.ac.kr (S.J.)

**Keywords:** animal movement, movement prediction, pattern prediction, predictive recurrent neural networks, kernel density image

## Abstract

Observing animal movements enables us to understand animal behavior changes, such as migration, interaction, foraging, and nesting. Based on spatiotemporal changes in weather and season, animals instinctively change their position for foraging, nesting, or breeding. It is known that moving patterns are closely related to their traits. Analyzing and predicting animals’ movement patterns according to spatiotemporal change offers an opportunity to understand their unique traits and acquire ecological insights into animals. Hence, in this paper, we propose an animal movement prediction scheme using a predictive recurrent neural network architecture. To do that, we first collect and investigate geo records of animals and conduct pattern refinement by using random forest interpolation. Then, we generate animal movement patterns using the kernel density estimation and build a predictive recurrent neural network model to consider the spatiotemporal changes. In the experiment, we perform various predictions using 14 K long-billed curlew locations that contain their five-year movements of the breeding, non-breeding, pre-breeding, and post-breeding seasons. The experimental results confirm that our predictive model based on recurrent neural networks can be effectively used to predict animal movement.

## 1. Introduction

Analyzing animal movements is the first step toward understanding the ecosystem. Animal movements provide potential information for obtaining ecological insights on topics such as habitat selection, population dynamics, and group behavior. Over the decades, many studies have been carried out based on the awareness of this importance [1,2,3,4]. Since the 1990s, the development of telemetry technologies such as the global positioning system (GPS) and advanced research and global observation satellite (ARGOS) has accelerated various studies to model animal movements. Furthermore, with the availability of remote sensing technology, diverse meteorological and geographical sensing data can be continuously acquired, and the amount is sufficient to carry out reasonable modeling. Many scientists have emphasized the need to use modeling processes to understand animal movements and the factors correlated with such movements [5,6,7,8].

Recently, as the versatility of machine learning methodologies has been observed in various domains, many efforts have been made to model animal movements using machine learning [9,10,11,12]. For instance, Patterson et al. proposed a two-phase prediction scheme to classify two animal behavior states: transient and resident. They used the maximum likelihood and monte carlo (MC) methods, and a hidden markov model (HMM) was used to predict the potential state of behavior change [13]. MoveHMM [14], which is a representative modeling tool based on hidden markov processes, showed that probability-based prediction of state switching is possible by using features such as animal step length and turning angle. More recently, the deep neural network (DNN) has become a major tool in animal movement modeling. For example, Hirakawa et al. focused on filling in the observation gap that frequently occurred while tracking wild animals periodically [15]. They found that previous interpolation methods (e.g., linear, curvilinear, random walk) are not sufficient to recover large spatial gaps. For this reason, they used inverse reinforcement learning (IRL) to recover the trajectory by estimating the gap as a reward function. Zhiping et al. utilized a six-layer convolution neural network (CNN) to distinguish fish shapes and track the zebrafish trajectory [16]. Browning et al. proposed a deep architecture to predict the diving behavior of seabirds associated with foraging. They combined the GPS and time depth record (TDR) from 108 individuals and trained deep learning models for predicting the behavior of European shags, common guillemots, and razorbills. An optimized model achieved 94% and 80% prediction accuracy of non-diving and diving behavior, respectively [17].

Despite the great effort to build animal movement models using geo records (e.g., GPS, ARGOS), the results have been limited to the identification of animal movement state or the analysis of correlations with environmental conditions. The key function that should be implemented in such a model is the prediction of animal movements according to changes in various spatiotemporal factors, which is possible by considering historical movement patterns. Such a model could be used for a variety of purposes, such as understanding animal behavior or protecting animal habitats, but to the best of our knowledge, there are very few reports on this topic. 

Therefore, in this paper, we propose an animal movement prediction scheme based on predictive recurrent neural networks (RNNs). To improve the prediction accuracy, we focus on three issues, which are our main contributions. (1) To fill the observation gap, we propose an optimized random forest-based interpolation scheme that considers weather information and movement-related vectors. In fact, interpolating the animal pathway is a very important task for predicting animal movement when considering realistic conditions. (2) We propose a method to construct an image sequence of representative animal movements by using the kernel density estimation (KDE) algorithm. The generated image sequence indicates a reasonable movement range of the animal. (3) We construct a predictive RNN model for movement prediction and evaluate its performance. 

## 2. Related Works

To date, many researchers have attempted to figure out the pattern of animal movement using various machine learning technologies. For instance, Jonsen et al. [18] proposed a meta-analysis method, which combines state-space models (SSM) and Bayesian approach for individual movement inference. They analyzed the relationship between leatherback turtle movement and environmental changes such as sea surface temperature. De Groeve et al. [19] developed an extracting method of spatiotemporal patterns in animal trajectories. They conducted a tree-based approach to identify the pattern of habitat selection and visualize the exploring temporal pattern of habitat use through hierarchical trees. Spigel et al. [20] proposed a conceptual simulation model of animal personality which includes foraging search performance, habitat preference, home ranging utilization pattern, social network and animal population progress. They argued that considering personality-dependent movement can generate far-reaching predictions about the spatial pattern in ecology. Wang [21] inferred animal behavior using machine learning methods such as state-space models, hidden Markov models, random forests, and support vector machines. Through various experiments, they confirmed that machine learning methods can be applied for quantifying the spatial and temporal distributions of specific behavior patterns. 

Predictions of long-short term animal movement and population shift have ecological importance because they are directly related to significant factors such as spatiotemporal changes of weather and habitat. Very few studies have predicted movement using long-short term tracking animal data. Li et al. [22] proposed a periodic movement analysis algorithm named ‘Periodica’. They focused on finding periods in complex animal movement and developed an optimal method of periodic animal behaviors analysis. The algorithm contains Fourier transform, autocorrelation, and hierarchical clustering for retrieving periods and formulating the periodic behaviors. Bar-David et al. [23] presented a simple recursion analysis model that simulates the movements of large-herbivore groups. They defined the movement pattern, which includes clockwise and counter-clockwise pattern and simply formulated the African buffalo trajectories using furrier transform. In 2010, Fink et al. [24] developed spatiotemporal exploratory models (STEM) using broad-scale survey data. They focused on the population change of animal distribution and proposed a flexible framework for analyzing the dynamic pattern of species occurrence and abundance from broad-scale data. To construct STEM, they utilized an ensemble model of decision trees. Through various experiments, they emphasized that STEM is a useful analytical tool of animal exploratory. Three years later, Fink et al. [25] presented an adaptive spatiotemporal exploratory model (AdaSTEM), which can exploit variation in the density of observations while adapting to multiple scales in space and time. The AdaSTEM utilized the crowdsourcing data of animal observation for building estimation model and provided an ecological insight that indicates population movement of long-range migration animals. Through AdaSTEM, they estimated the spatiotemporal distribution of Barn Swallow across the western hemisphere during the breeding season, fall migration and winter migration.

To summarize, previous studies on movement prediction models have mainly focused on the behavior state changes such as ‘breeding’ to ‘migrating’ or ‘roaming’ to ‘resting’. As far as we know, few works have been done to predict long-short term animal movement or population shift especially by using continuous tracking data to construct a reasonable prediction model. In this paper, we propose an animal movement prediction model to predict the aggregate movements of animals over short-long term periods by considering continuous tracking data of individuals which are effected by spatiotemporal changes such as weather and movement characteristics.

## 3. Methods

**Figure 1:**
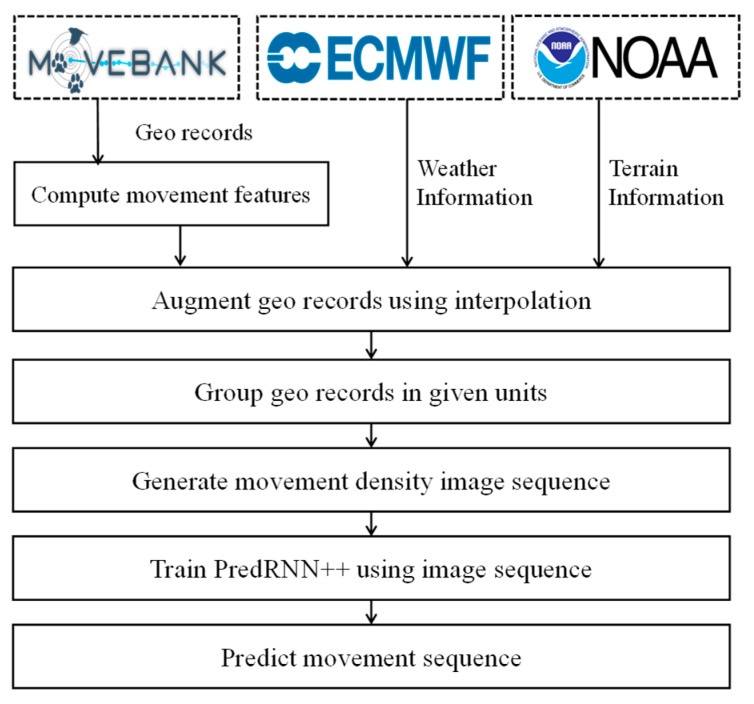
Overall flow of animal movement prediction.

Figure 1 presents the overall flow of our animal movement prediction scheme. First, we collect and investigate the spatiotemporal data closely related to animal movements, such as geo records of animals, weather, and terrain data. In order to refine the movement pattern, we perform random forest interpolation by considering collected data as independent feature values. Then, we uniformly split the geo records and generate the movement density sequence that represents the valid range of animal movement. To build a prediction model, we train a predictive RNN using movement density sequences. Lastly, we evaluate the prediction performance of the model based on various accuracy metrics and visualize the prediction results. 

### 3.1. Dataset

For the collection of animal movement data, we used geo records of animals from the Movebank website [26]. Movebank is an online database of animal tracking data. In particular, we used the “Long-billed curlew migration from Idaho” dataset, which contains 64 trajectories of long-billed curlews. The dataset was collected over six years, from 2013 to 2019, and it consists of ARGOS latitude, ARGOS longitude, timestamp, and tag local identifier. According to the dataset description, the number of deployed locations is 148,983, and there are no outliers. The coordinate system follows the WGS84 reference format.

**Figure 2:**
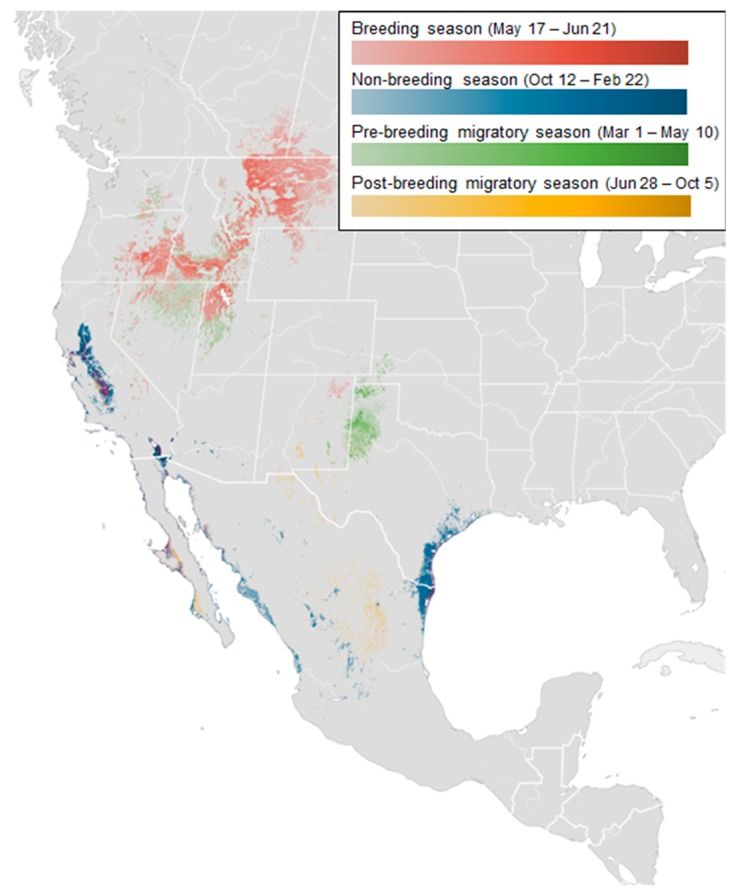
Seasonally averaged abundance map of long-billed curlews.

To represent the movement range of long-billed curlews, we use the abundance map [27]. Figure 2 shows the abundance distribution map of long-billed curlews. In the map, the four seasons are represented by different colors, and the distribution density is expressed by the contrast of color. The distribution itself is calculated based on geo records from individuals that have moved more than one kilometer over one hour. For instance, the dark red indicates high-density areas during the breeding season (e.g., Montana, Idaho). During the non-breeding season, San Francisco and California show high density. 

Animal migration routes are closely related to weather and terrain. Hence, for prediction, we collected weather and terrain data from the European Centre for Medium-Range Weather Forecasts (ECMWF) and the US National Oceanic and Atmospheric Administration (NOAA) and selected eight features for our feature set: Humidity, vertical wind speed, horizontal wind speed, temperature, relative humidity, cloud coverage, land classification label (GlobCover), and land–sea mask. 

In this paper, we use four typical features to represent animal movements: Heading, speed, acceleration, and moving distance. Such movement-related features can be easily calculated from the collected time-location records by using the “move” package of R statistics [28]. 

### 3.2. Movement Interpolation

Despite the high quality of tracking data, GPS location accuracy and positioning success (ratio between the observed and expected number of locations) are negatively influenced by a number of factors, including the terrestrial atmosphere, satellite constellation, environment of the transmitters (habitat, topography, and weather), and behavior pattern and movement intensity of the tagged animal [29]. When analyzing animal trajectories, lack of observation or erroneous geo records lead to inaccurate results. Hence, it is essential to estimate missing or erroneous animal locations based on correct geo records, which is called movement interpolation. Various interpolation methods, such as linear, cubic spline, and polynomial interpolation, have been widely used in studies on animal movement. Table 1 shows the major interpolation methods.

All these methods perform interpolation using a few observation points. Since they do not consider the environmental features such as weather and terrain, the interpolation accuracy could be lowered. To alleviate this problem, we use the random forest method, which can consider both observation points and environmental factors. The random forest method works by constructing several weak decision trees, which are trained by a random subset of features, and producing the result by averaging or voting all individual trees. The basic principle of random forest is bootstrap aggregating (Bagging) to reduce the variance of predicted values. Bagging selects the specific number of training set randomly and fits for optimizing tree construction. For this reason, the random forest has excellent performance in classification and regression problems. 

**Figure 3:**
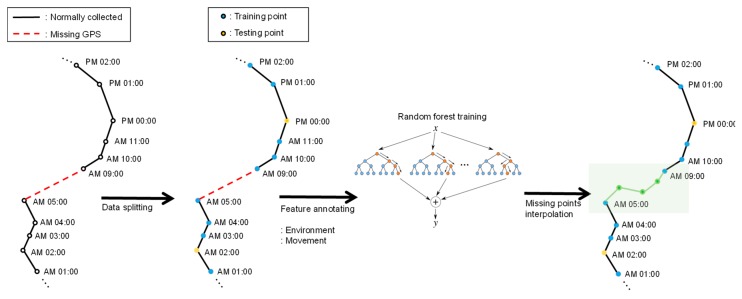
Movement interpolation using the random forest method.

Figure 3 shows an example of movement interpolation process. Two normally observed points are connected by a solid line. If there are any missing points, then they are connected by a red dotted line. For instance, in the figure, we can see that observation records are missing from 6:00 am to 8:00 am. To interpolate these missing records, we first split the geo records into training sets and test sets. Then, we generate a feature tuple for each observation record by augmenting environmental data and movement data as we described in Section 3.1. Each feature tuple is in the form of $p_{i}=\left\{lat_{i},\;long_{i},\;time_{i},heading_{i},speed_{i},\;acc_{i},\;md_{i},\;hum_{i},\;relhum_{i},\;vwind_{i},hwind_{i},\;temp_{i},\;cloud_{i},\;land_{i},\;landseamask_{i}\right\}$. For a time sequence of length *i*, we can make a feature set *P* of $\left\{p_{1},\;p_{2},\;\ldots ,\;p_{i}\right\}$. Basically, we select missing time points at random in the interpolation. Also, we assume that the points of test set are equal to missing time points. Our random forest model interpolates missing bivariate location (latitude and longitude) using a feature tuple of previous missing time points. For example, if a missing location exists a certain time *t* ($p_{t}\in$ *P*), we predict the missing bivariate location at time *t* using the feature tuple at time *t*-1 as independent variables. The random forest training process follows bagging algorithm, which randomly selects a sample of size *k* from the feature set *P* and fits the decision trees. During the tree evolution, each node of the tree chooses the best split size given a randomly selected sample. Each tree is grown to the maximum size until it has no longer split. After tree evolution, prediction for missing coordinate can be calculated by averaging the predictions from all trees. Once the training is over, we are given an optimized random forest model, which can be used for interpolating missing observation points. After training, we perform inbuilt cross-validation and calculate the prediction accuracy using mean absolute percentage error (MAPE) and root mean square error (RMSE) to obtain an optimal random forest. In particular, we used the grid search method to find optimal parameters for the random forest. To construct and train the model, we used scikit-learn [34], a python machine learning package. 

### 3.3. Movement Density Sequence Generation

**Figure 4:**
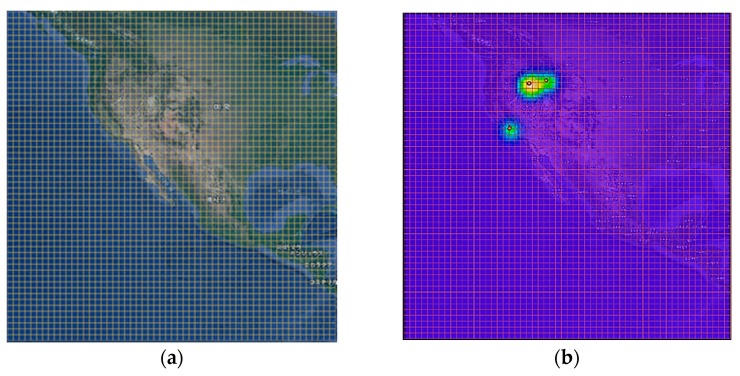
Example of movement density image construction—(**a**) grid generation; (**b**) kernel density generation.

After movement interpolation, we generate the movement density sequence that eventually indicates the movement range of animals. The movement density gives information which represents the spatial distribution of the moving animals. First, we construct a grid map of *I* × *J* considering the region of interest. *I* and *J* indicate the image width and height, respectively. Figure 4a shows an example of *I* × *J* grid map. 

To describe animal movements effectively, we use the KDE method. This method has been widely used in movement ecology and detection of animal habitat [35,36,37]. It is one of non-parametric density estimation methods and improves the non-zero probability problem of the histogram method by using the kernel function. A kernel function *K* is controlled by the bandwidth parameter *h*. According to the kernel definition, we calculate the probability density function *f* for the input geo records using the following equation.

(1) $${\hat{f}}_{h}\left(y\right)=\;\frac{1}{nh}\overset{n}{\underset{i=1}{\sum }}K\left(\;\frac{y-x_{i}}{h}\;\right)$$

here, *n* and *x_i_* indicate the number of geo records and geo record at time *i*, respectively, and *y* indicates estimated density value. In particular, to reduce the complexity of computation, we use the Gaussian kernel for generating density map. In addition, we use the bandwidth parameter of 5 and normalize the kernel values to a range of 0 to 1. Figure 4b shows an example of animal movement density map using KDE. 

### 3.4. Movement Prediction

In this section, we describe how to construct a movement prediction model using predictive RNNs. The predictive RNN structure is known to give better accuracy for sequence-based prediction than single or shallow structures. Predictive RNN structure such as convolutional long short-term memory (LSTM) has a stack of learning and memory units, which is effective in solving sequence prediction problems [38,39]. For instance, Xingjian et al. introduced a convolutional LSTM network approach for precipitation nowcasting. Using the Radar Echo dataset, which has a sequence of weather satellite images, they constructed a prediction model using the continuous stack of convolution LSTMs [40].

**Figure 5:**
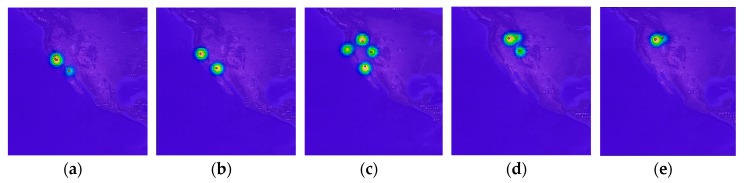
Examples of movement density sequence–(**a**) 12 Dec. 2013 to 12 Jan. 2014; (**b**) 12 Jan. 2013 to 12 Feb. 2014; (**c**) 12 Feb. 2014 to 12 Mar. 2014; (**d**) 12 Apr. 2014 to 12 May 2014; (**e**) 12 May 2014 to 12 Jun. 2014.

Likewise, we consider the movement prediction problem as a sequence-to-sequence prediction. For the animal movement observation period *k*, we can define a sequence of movement densities of size *T* defined by the input size and *k* by $X^{k}=\left\{X_{1}^{k},X_{2}^{k}\ldots ,\;X_{T}^{k}\right\}$. Usually, each $X_{i}^{k}$ has a centroid of movement ranges, and the centroid shows the maximum density value of KDE. Generally, for a given input sequence $\left\{X_{T-\tau }^{k},X_{T-\tau +1}^{k}\ldots ,\;X_{T}^{k}\right\}$, our model predicts its movement density sequence $\left\{X_{T+1}^{k},X_{T+2}^{k}\ldots ,\;X_{T+\tau }^{k}\right\}$. Figure 5 illustrates an example of $X_{T}^{k}$ where the observation period *k* is one month.

**Figure 6:**
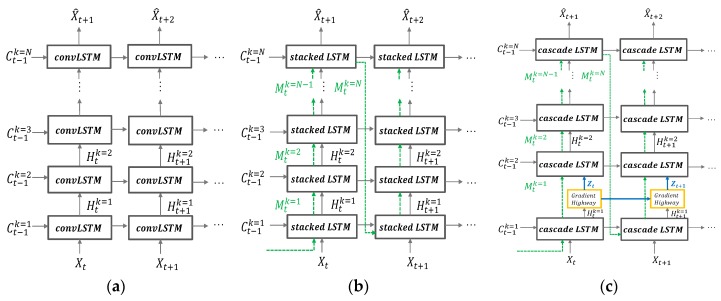
Structural differences of (**a**) convolutional stacking LSTM; (**b**) PredRNN; (**c**) PredRNN++.

To predict animal movements, we use the PredRNN++ model [41]. RNN architecture has been widely used in the sequence data prediction. However, standard RNN can’t handle long-term temporal dependency because its loss gradient deteriorates exponentially over time. Despite convolutional LSTM being suggested as an alternative to standard RNN, it showed the vanishing gradient problem. PredRNN++ differs from the convolutional LSTM in terms of internal structure and mechanism of gradient transition to solve the back propagation problem. Figure 6 compares the structural differences of convolutional stacking LSTM, PredRNN [42], and PredRNN++. 

PredRNN and PredRNN++ have in common that they use a spatiotemporal memory transient concept, but PredRNN++ differs from PredRNN in that it has unique structures of increasing recurrent depth. PredRNN++ has a cascade spatiotemporal memory that is effective in analyzing spatial correlation. Its cascade LSTM unit has a dual memory form of temporal memory and spatial memory. The spatial memory, $M_{t}^{k},$ depends on $M_{t-1}^{k}$ from the transient path, which is represented by dotted lines in Figure 6c. The temporal memory $C_{t}^{k}$ depends on previous state $C_{t-1}^{k}$ and is adjusted by current forget, input and input modulation gate. Here, k indicates the vertical depth of the layer. Another difference is the existence of the gradient highway unit (GHU), which prevents quick vanishing and can send the gradient information to the deeper layer. The advantages of GHU were confirmed through various experiments [41].

**Figure 7:**
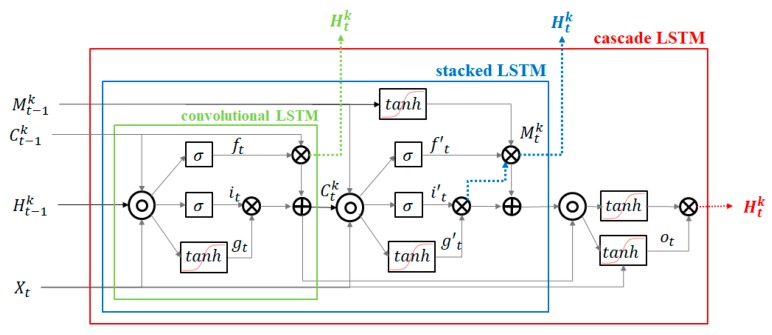
Structural differences of convolutional LSTM, stacked LSTM, and cascade LSTM.

Figure 7 shows the structural comparison of convolutional LSTM, stacked LSTM, and cascade LSTM. The green, blue, and red boxes indicate standard convolutional LSTM, stacked LSTM, and cascade LSTM, respectively. The element notations of structures are as follows: The sequence input at time t (which denotes $X_{1},\;\ldots \;X_{t})$, output state $C_{1}^{k},\;\ldots \;C_{t}^{k}$, hidden state $H_{1}^{k}\;\ldots \;H_{t}^{k}$, spatiotemporal memory $M_{t}^{k}$ input gates $i_{t}$, forget gates $f_{t}$, input modulation gate $g_{t}$, and output gate $o_{t}$. As shown in Figure 7, to obtain effectiveness of sequence modeling, the cascade LSTM adds non-linear hyperbolic tangent layers to recurrent transitions more than convolutional LSTM and stacked LSTM. This has the effect of increasing the network depth, so it can efficiently predict images or video where sudden changes occur. The green-marked $H_{t}^{k}$, blue marked $H_{t}^{k}$ and red $H_{t}^{k}$ are desired output of convolutional LSTM, stacked LSTM, and cascade LSTM respectively. The final output of cascade LSTM, $H_{t}^{k}$, is more suitable for non-linearity data processing and prediction than convolutional LSTM and stacked LSTM outputs. Since most animal movements have non-linearity, the structure of the cascade LSTM is useful for predicting animal movements.

(2) $$\left(\begin{array}{c} g_{t}\\ \begin{array}{c} i_{t}\\ f_{t} \end{array} \end{array}\right)=\left(\begin{array}{c} tanh\\ \begin{array}{c} \sigma \\ \sigma \end{array} \end{array}\right)W_{1}*\left[X_{t},\;H_{t-1}^{k},\;C_{t-1}^{k}\right]$$

(3) $$C_{t}^{k}=f_{t}\;⊙\;C_{t-1}^{k}+\;i_{t}\;⊙\;g_{t}$$

(4) $$\left(\begin{array}{c} g{{}^{\prime }}_{t}\\ \begin{array}{c} i{{}^{\prime }}_{t}\\ f{{}^{\prime }}_{t} \end{array} \end{array}\right)=\left(\begin{array}{c} tanh\\ \begin{array}{c} \sigma \\ \sigma \end{array} \end{array}\right)W_{2}*\left[X_{t},\;C_{t}^{k},\;M_{t}^{k-1}\right]$$

(5) $$M_{t}^{k}=f{{}^{\prime }}_{t}\;⊙\;\tan h\left(W_{3}*M_{t}^{k-1}\right)+\;i{{}^{\prime }}_{t}\;⊙\;g{{}^{\prime }}_{t}$$

(6) $$o_{t}=\left(W_{4}*\left[X_{t},\;C_{t}^{k},\;M_{t}^{k}\right]\right)$$

(7) $$H_{t}^{k}=o_{t}\;⊙\;\tan h\left(W_{5}*\left[C_{t}^{k},\;M_{t}^{k}\right]\right)$$

Equations (2)–(7) present the operating of cascade LSTM, where ∗ and ⊙ indicates the operator of the convolution and the Hadamard product, σ is the Sigmoid function. The square brackets present concatenation of each tensor. The PredRNN++ used convolutional filters $W_{1\sim 5}$, where $W_{3}$ and $W_{5}$ has one by on convolution filer. The basic equations derived from standard LSTM [43] and the difference of standard LSTM and PredRNN++ is number of hyperbolic tangent layers and existing spatiotemporal memory cells. Equations (2) and (3) present standard LSTM gates operating equations, and Equation (4) is spatiotemporal gates update equation regulated by previous spatiotemporal memory, $M_{t-1}^{k}$, and current state, $C_{t}^{k}$. Equations (5) and (6) shows current spatiotemporal memory, $M_{t}^{k}$, update operation. Final output, $H_{t}^{k}$, is computed based on dual memory states $C_{t}^{k}$ and $M_{t}^{k}$. 

### 3.5. Model Construction

In this section, we describe how to construct our prediction model using PredRNN++. To train PredRNN++, we first set the resolution of the input sequence to 224 × 224 based on prediction accuracy and computation time. In addition, we use a 5-layer cascade LSTM with 128, 128, 64, 64, and 64 channels, which is known to give the best result. Here, the second layer corresponds to GHU in Figure 6. In addition, we use ADAM optimizer with $10^{-3}$ learning rate and convolution filter size of 5. Following the training strategy of the predictive neural network [41,42], we use the scheduled sampling strategy. The sampling strategy conditionally selects true sequence and predicted values of the model as training input. It prevents initial learning problem and cover the inconsistency between prediction and training. For training, we used 80% of the complete dataset which were recorded from 10 April 2013 to 15 November 2017 as training and validation set, and the remaining 20% which were recorded from 16 November 2017 to 14 May 2019 as test set. 

## 4. Experiments

### 4.1. Experiment Designs

**Figure 8:**
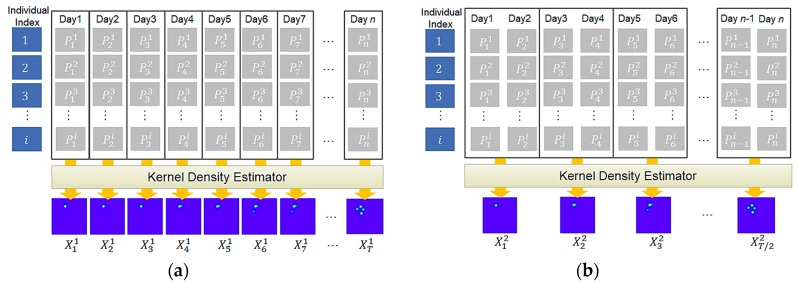
Examples of input sequence generation–(**a**) example of case 1; (**b**) example of case 2.

To predict animal movement over time, we first need to generate a sequence of kernel density images from collected geo records. In the experiment, we considered 10 different cases depending on how to group geo records, how many days to consider as input, and how many days to predict as output. For instance, in Figure 8, $P_{i}$ represents one collected geo record and i indicates an individual of a particular animal species, *E*. For each group of geo records, the KDE generates one density image. For instance, Figure 8a,b shows a sequence of density images generated for a sequence of daily and two-day geo records, respectively. To see the effect of grouping size, we consider five different grouping sizes, which are 1, 2, 3, 7, and 15. After generating movement density images, we use them as an input for our predictive RNN. Table 2 shows an outline of the experiments.

The density image has 224 × 224 resolution and corresponds to the rectangle area on the map according to WGS84 system with coordinates (–82,56), (–138,56), (–82,0), and (–138,0). For the generation of movement density sequence, we used the “SpatialEco” package [44], which is implemented in the R Studio software. Using the generated density images, we trained the PredRNN++.

### 4.2. Random Forest Interpolation

To compare the performances of interpolation methods, we measure the MAPE and RMSE. In statistics, MAPE is a popular metric for estimating prediction accuracy. MAPE and RMSE are defined by Equations (8) and (9), respectively. Here, n is the number of geo records, and $Y_{i}$, and ${\hat{Y}}_{i}$ are the actual and predicted values, respectively.

(8) $$MAPE=\frac{100}{n}\overset{n}{\underset{i=1}{\sum }}\left|\frac{Y_{i}-{\hat{Y}}_{i}}{Y_{i}}\right|$$

(9) $$RMSE=\sqrt{\frac{\sum_{i=1}^{n}{\left(Y_{i}-{\hat{Y}}_{i}\right)}^{2}}{n}}$$

We consider four interpolation methods as baseline, which are linear, Bezier, cubic spline, and kinematic, and compare them with our random forest method. In the test for comparing them, we first selected 15 representative individuals that include typical movement patterns such as resting, foraging and migration. Then, we built 15 random forest interpolation models for each individual. To construct such random forests, we selected 80% of individual geo records as training set and 20% of individual geo records as test set. When measuring the MAPE and RMSE, we considered actual geo records in the test set as missing and evaluated how multivariate random forest model recover well the missing values. Response variables are longitude and latitude at time *i* which are estimated from *i*-1 feature tuples. Table 3 and Table 4 show the MAPE of longitudes and latitudes. Table 5 and Table 6 show the RMSE of longitudes and latitudes. In the tables, the best MAPE and RMSE values are marked in bold for each individual. In most cases, our random forest method performed well and the average accuracy was the best. 

### 4.3. Movement Prediction

To evaluate the prediction performance of animal movements, we used three different metrics: mean square error (MSE), structural similarity index (SSIM), and Gaussian mixture centroid distance. MSE is a standard method for evaluating the prediction quality in statistics; it measures the average square difference between pixel values of ground truth image *G* and pixel values of predicted image *P* by using Equation (10). In the equation, *N* × *M* is the total number of pixels and (*i*,*j*) represents a pixel of image.

(10) $$MSE\left(G,\;P\right)=\;\frac{1}{NM}\overset{N}{\underset{i=1}{\sum }}\overset{M}{\underset{j=1}{\sum }}{\left[P\left(i,j\right)-G\left(i,j\right)\right]}^{2}$$

On the other hand, SSIM is a method for measuring the similarity between two images and is designed to improve on traditional methods such as peak signal-to-noise ratio (PSNR) and MSE. SSIM is calculated using Equation (11), where μ, σ, and σ*_GP_* are the average of pixels, variance, and covariance of *G* and *P*, respectively. By setting the range of SSIM from −1 and 1, we consider that, for the two images, there exists higher structural similarity, as their SSIM values are close to 1.

(11) $$SSIM\left(G,\;P\right)=\;\frac{\left(2\mu_{G}\mu_{P}\right)\left(2\sigma_{GP}+c_{2}\right)}{\left(2\mu_{G}^{\;2}+\mu_{P}^{\;2}+c_{1}\right)\left(\sigma_{G}^{\;2}+\sigma_{P}^{\;2}+c_{2}\right)}$$

In the ecology research field, centroid analysis is widely used for evaluating animal dynamics [45,46,47]. Since our movement prediction model produce a movement sequence as prediction, we can calculate the density of movements and their centroids. We measure the centroid distance between the input image, *G*, and predicted image *P*, which indicates how accurate our model is in the prediction of animal movements. For measuring centroid distance, we first apply Gaussian mixture fitting [48] to extract centroids from each image and calculate their real coordinates. Then, we calculate their distance using the real coordinates. As mentioned above, we consider ten different cases in the experiment by grouping unit, input days, and output days and calculated the average for each metric. Table 7 shows the results, in which our prediction model showed the best performance from case 1. 

To evaluate the performance of our model, we compare it with vector auto regressive model (VAR), which is known to be useful for time-series prediction. In the comparison, we calculate the movement centroids using the Gaussian mixture fitting and use them as input values of the VAR. To construct VAR, we used the R package ‘VARS’ and utilized the ‘VARselect’ function to find out the most appropriate setting for VAR fitting. The comparison results are as follows. 

From these Table 8, Table 9 and Table 10, we can see that our model outperforms VAR in most cases. In particular, our model, when trained using movement patterns of short observation period, showed better performance. In summary, our model can cover long range and short range predictions and hence can be used to predict not only seasonal movements such as strong migration but also typical movements such as foraging and roaming.

**Figure 9:**
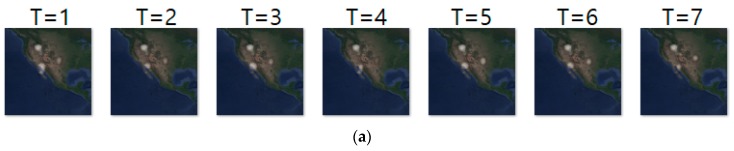

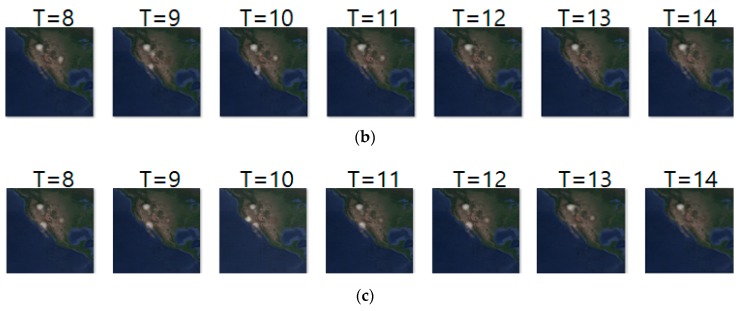
Movement prediction result in case 1–(**a**) input sequence; (**b**) ground truth; (**c**) predicted sequence.

Figure 9 shows an example of movement prediction. In the figure, for an input sequence in (a), (b) and (c) show the ground truth sequence and predicted sequence, respectively. The input and ground truth sequences represent the short-distance migration movement of long-billed curlews from 10 May 2018 to 23 May 2018. The short-distance migration occurs between pre-breeding season and breeding season. This figure demonstrates that our model predicts the density location of animal movements after one-day (T = 1) with an error range of approximately 23 km. That is, based on the density images of the last seven days, our prediction model generated the density images of next seven days. To analyze the prediction accuracy in more detail, we conducted daily comparison of RMSE, SSIM, and centroid distance results are shown in Table 11. In the table, we can see that overall prediction results are quite reasonable and that prediction for the first day is the best. 

**Figure 10:**
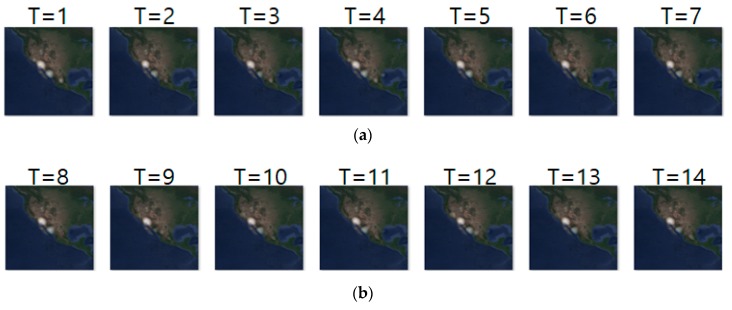

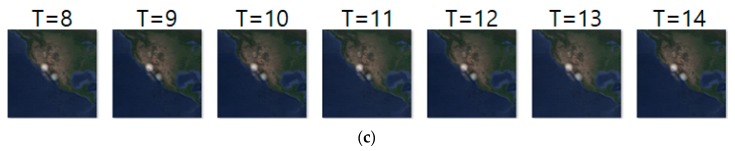
Movement prediction result–(**a**) input sequence; (**b**) ground truth; (**c**) predicted sequence.

Figure 10 shows another prediction example for case 2, where we analyze geo records over two days. The input and ground truth sequences represent non-breeding season movements of long-billed curlews from 13 October 2018 to 8 November 2018. Generally, long-billed curlews have a habit of not deviating greatly from the habitat at the non-breeding season. Density images in Figure 10 shows such movements at the non-breeding season. Table 12 shows their prediction result. Compared with Table 11, most metrics gave worse results since case 2 considered geo-records using a longer period (of two days).

**Figure 11:**
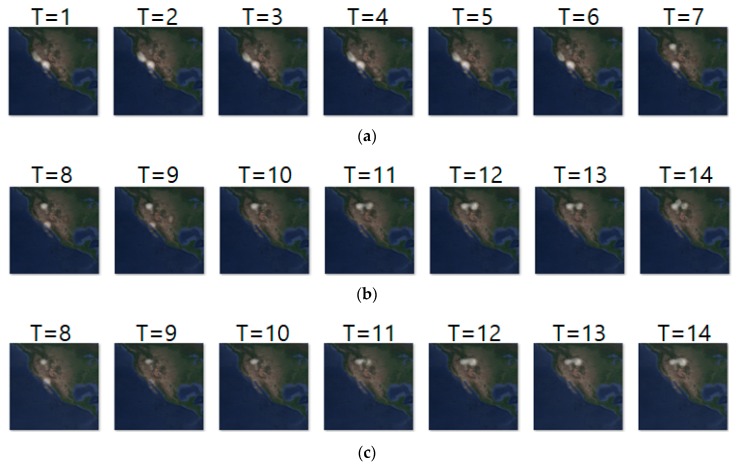
Movement prediction result–(**a**) input sequence; (**b**) ground truth; (**c**) predicted sequence.

Likewise, Figure 11a–c shows the input sequence, ground truth sequence and predicted sequence of case 4, respectively. The input and ground truth sequences indicate the migration movement of long-billed curlews from 7 February 2018 to 9 May 2018. In the case 4, we generated one movement density image for geo records of seven days. In the figure, we can see three states of migration: non-breeding season movement shown at T = 1 to 6, migration beginning shown at T = 7 to 10 and migration ending shown at T = 11 to 14. Table 13 shows the daily comparison of RMSE, SSIM, and centroid distance. 

**Figure 12:**
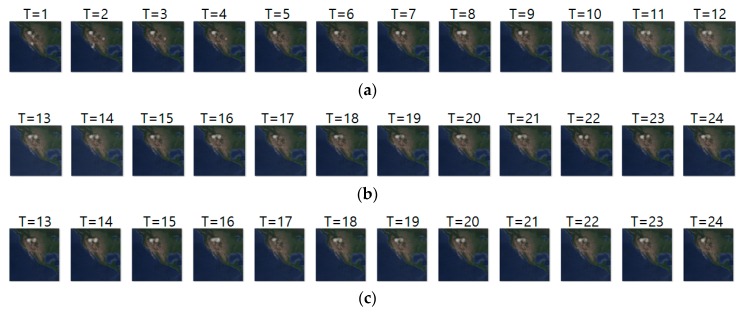
Movement prediction result–(**a**) input sequence; (**b**) ground truth; (**c**) predicted sequence.

Figure 12a–c shows the input sequence, ground truth and predicted sequence of case 6, respectively. The data we used for case 6 are migration movements of long-billed curlews from 7 March 2018 to 30 March 2018. As we showed in the Figure 12, long-billed curlews undertook pre-breeding and breeding migration. We can see that the image at T = 1 shows the density at the pre-breeding season, the next seven images show the migration beginning movements, and the remaining images show migration ending movements. Table 14 shows daily comparison of RMSE, SSIM, and centroid distance in case 6. Notably, when the animals showed static movements, our model showed a stable prediction performance of less than 90 km error. 

**Figure 13:**
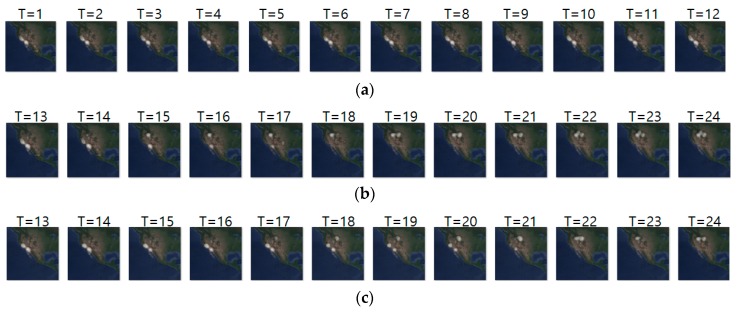
Movement prediction result–(**a**) input sequence; (**b**) ground truth; (**c**) predicted sequence.

Figure 13a–c shows the input sequence, ground truth, and predicted sequence of case 9, respectively. The input and ground truth sequences show the migration movement of long-billed curlew from 16 November 2017 to 3 May 2018. In Figure 13a,b, we can see the starting migration movement at T = 14, pre-breeding migratory and migration for breeding at T = 15 to 18, and migration ending at T = 19 to 24. Table 15 shows daily comparison of RMSE, SSIM, and centroid distance in case 9. In this experiment, the input sequence showed a static movement and the ground truth sequence showed a dynamic movement pattern from T = 15 to 18. However, our model did not predict such dynamic movement precisely.

**Figure 14:**
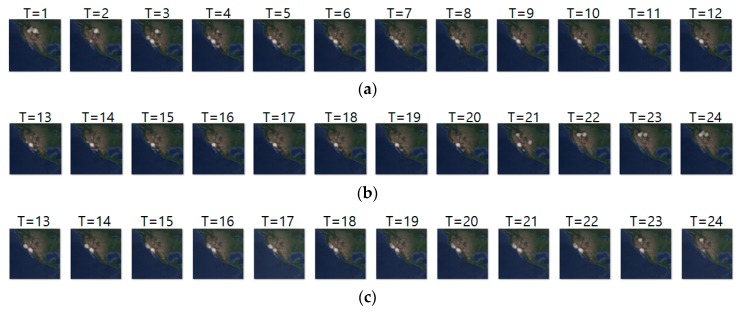
Movement prediction result–(**a**) input sequence; (**b**) ground truth; (**c**) predicted sequence.

Figure 14a–c shows the input, ground truth, and predicted sequences of case 10, respectively. The input and ground truth sequences are the migration movements of long-billed curlews from 19 May 2018 to 14 May 2019. We used the 15-day geo records to make a one-movement density image and as a sequence to predict the migration pattern for one year. As shown in Figure 14a,b, we observe that non-breeding season migration was shown at T = 1 to 3 and T = 21 to 24, and breeding season movement was shown at T = 4 to 20. Table 16 shows the daily comparison of RMSE, SSIM, and centroid distance of case 10. The result shows that there are relatively low distance errors at T = 13 to 20, but as migration starts at T = 21 to 33, we can see relatively high distance errors. 

## 5. Conclusions

In this paper, we proposed a novel approach for predicting animal movements using a predictive RNN. For accurate movement prediction, we first showed how to compensate for any missing geo records using random forest regression. To interpolate the missing geo records, we used random forest based on animal movement features and environmental features such as terrain and weather. Compared with other popular interpolation methods, our proposed model achieved higher interpolation accuracy. After augmenting geo records, we grouped geo records by various units and created a sequence of movement density images, which represent movement trends of animals. Then, we built a movement prediction model by training PredRNN++ using these sequence data. We evaluated the model’s performance through various experiments. The results showed that our model is quite effective for animal movement prediction. 

## Figures and Tables

**Table 1:** Comparison of major interpolation methods.

Interpolation Method	Advantages	Disadvantages	Reference(s)
Linear	Quick calculation No overshooting No undulation	Inaccurate interpolation (Non-linear movement)	Wentz et al. [30] (2003)
Cubic spline	Stable Less computation	Overshooting problem	Yu et al. [31] (2003)
Polynomial	Simple calculation	Expensive computing Undulation problem	Tremblay [32] (2005)
Bezier	Simple calculation Effective interpolation (Non-linear movement)	Sampling points decision problem	Tremblay [32] (2005)
Kinematic	Effective interpolation (Fast-moving or linear movement)	Inaccurate interpolation (Large spatial movement)	Long [33] (2016)

**Table 2:** Experiment outline.

Prediction Cases	No. of Days to Group Geo Records	No. of Input Images	No. of Predicted Images
Case 1	1	7	7
Case 2	2	7	7
Case 3	3	7	7
Case 4	7	7	7
Case 5	15	7	7
Case 6	1	12	12
Case 7	2	12	12
Case 8	3	12	12
Case 9	7	12	12
Case 10	15	12	12

**Table 3:** Comparison of MAPEs for missing longitude records interpolation.

Interpolation Case #	MAPE (longitude)				
	Linear	Bezier	Cubic Spline	Kinematic	Random Forest
Individual #1	**0.025**	0.052	0.030	0.715	0.030
Individual #2	0.044	0.038	0.063	0.684	**0.040**
Individual #3	0.010	0.011	0.012	0.689	**0.002**
Individual #4	**0.038**	0.095	0.048	0.744	0.159
Individual #5	0.058	0.101	0.067	0.713	**0.01**
Individual #6	0.035	0.085	0.061	0.752	**0.032**
Individual #7	0.017	0.036	0.019	0.699	**0.001**
Individual #8	0.030	0.177	0.049	0.699	**0.008**
Individual #9	0.010	0.038	0.012	0.732	**0.006**
Individual #10	0.042	0.039	0.052	0.714	**0.013**
Individual #11	0.031	0.067	0.049	0.736	**0.005**
Individual #12	0.035	0.034	0.042	0.746	**0.012**
Individual #13	**0.066**	0.086	0.103	0.768	0.076
Individual #14	0.044	0.040	0.057	0.776	**0.034**
Individual #15	**0.010**	0.027	0.019	0.835	0.016
Avg.	0.033	0.062	0.046	0.733	**0.030**

**Table 4:** Comparison of MAPEs for missing latitude records interpolation.

Interpolation Case #	MAPE (latitude)				
	Linear	Bezier	Cubic Spline	Kinematic	Random Forest
Individual #1	**0.132**	0.462	0.177	2.449	0.180
Individual #2	**0.082**	0.132	0.118	2.088	0.181
Individual #3	0.049	0.038	0.059	2.519	**0.026**
Individual #4	0.256	0.302	0.263	2.946	**0.179**
Individual #5	0.246	0.453	0.303	2.315	**0.231**
Individual #6	**0.145**	0.686	0.377	2.592	0.301
Individual #7	**0.051**	0.130	0.068	2.517	0.065
Individual #8	**0.068**	1.275	0.228	2.439	0.124
Individual #9	**0.019**	0.263	0.044	2.762	0.046
Individual #10	0.235	**0.150**	0.253	2.368	0.159
Individual #11	0.070	0.148	0.136	2.974	**0.047**
Individual #12	0.084	0.125	0.136	2.714	**0.102**
Individual #13	0.393	0.487	0.701	3.209	**0.254**
Individual #14	0.153	0.076	0.164	2.537	**0.048**
Individual #15	**0.043**	0.070	0.086	2.889	0.052
Avg.	0.135	0.320	0.207	2.621	**0.133**

**Table 5:** Comparison of RMSEs for missing longitude records interpolation.

Interpolation Case #	RMSE (longitude)				
	Linear	Bezier	Cubic Spline	Kinematic	Random Forest
Individual #1	**0.067**	0.230	0.087	0.880	0.106
Individual #2	0.115	0.081	0.156	0.854	**0.094**
Individual #3	0.024	0.016	0.024	0.815	**0.003**
Individual #4	0.188	0.455	**0.144**	0.828	0.465
Individual #5	0.270	0.511	0.271	0.916	**0.022**
Individual #6	**0.158**	0.280	0.192	0.818	0.352
Individual #7	0.028	0.054	0.033	0.826	**0.003**
Individual #8	0.063	0.725	0.140	0.832	**0.030**
Individual #9	0.025	0.243	0.029	0.865	**0.021**
Individual #10	0.188	0.070	0.191	0.896	**0.024**
Individual #11	0.048	0.110	0.074	0.824	**0.007**
Individual #12	0.130	0.062	0.123	0.966	**0.035**
Individual #13	0.287	0.293	0.328	0.843	**0.256**
Individual #14	0.105	0.091	0.158	0.971	**0.104**
Individual #15	**0.018**	0.048	0.038	1.008	0.048
Avg.	0.114	0.218	0.133	0.876	**0.105**

**Table 6:** Comparison of RMSEs for missing latitude records interpolation.

Interpolation Case #	RMSE (latitude)				
	Linear	Bezier	Cubic Spline	Kinematic	Random Forest
Individual #1	**0.233**	0.821	0.273	0.932	0.385
Individual #2	**0.078**	0.178	0.104	0.866	0.199
Individual #3	0.078	0.017	0.068	0.811	**0.012**
Individual #4	0.462	0.503	0.33	0.914	**0.283**
Individual #5	0.430	0.745	0.464	1.054	**0.224**
Individual #6	**0.243**	0.872	0.392	0.859	0.566
Individual #7	**0.023**	0.053	0.033	0.825	0.040
Individual #8	**0.047**	1.799	0.308	0.859	0.205
Individual #9	**0.012**	0.729	0.078	0.863	0.127
Individual #10	0.548	0.074	0.549	0.978	**0.136**
Individual #11	0.027	0.072	0.107	0.926	**0.018**
Individual #12	**0.054**	0.073	0.105	1.407	0.085
Individual #13	0.493	0.437	0.574	0.913	**0.278**
Individual #14	0.317	0.046	0.244	0.923	**0.036**
Individual #15	**0.022**	0.038	0.052	0.923	0.064
Avg.	0.204	0.430	0.245	0.937	**0.177**

**Table 7:** MSE, RMSE, SSIM, and centroid distance measure results.

	**Evaluation Metrics**				
	**Avg. MSE**	**Avg. RMSE**	**Avg. SSIM**	**Avg. Centroid Distance (in pixels)**	**Avg. Centroid Distance (in km)**
Case 1	44.236	6.651	0.970	4.583	110.179
Case 2	44.571	6.676	0.965	5.299	127.413
Case 3	51.090	7.148	0.963	5.586	134.299
Case 4	58.142	7.625	0.958	7.028	168.983
Case 5	72.445	8.511	0.937	8.492	204.182
Case 6	43.445	6.591	0.968	4.698	112.944
Case 7	43.182	6.571	0.962	5.037	121.099
Case 8	53.219	7.295	0.953	5.487	131.933
Case 9	62.847	7.928	0.943	7.602	182.779
Case 10	142.449	11.935	0.932	10.265	246.795

**Table 8:** Comparison results of Case 1, Case 6 and VAR.

	Evaluation Metrics		
	RMSE (in Centroid Pixel)	Avg. Centroid Distance (in pixels)	Avg. Centroid Distance (in km)
Case 1	**6.872**	**4.583**	**110.179**
Case 6	6.914	4.698	112.944
VAR	10.573	12.840	308.695

**Table 9:** Comparison results of Case 4, Case 9 and VAR.

	Evaluation Metrics		
	RMSE (in Centroid Pixel)	Avg. Centroid Distance (in pixels)	Avg. Centroid Distance (in km)
Case 4	**7.885**	**7.328**	**176.191**
Case 9	8.021	7.602	182.779
VAR	9.075	11.0211	264.957

**Table 10:** Comparison results of Case 5, Case 10 and VAR.

	Evaluation Metrics		
	RMSE (in Centroid Pixel)	Avg. Centroid Distance (in pixels)	Avg. Centroid Distance (in km)
Case 5	**8.846**	**8.492**	**204.182**
Case 10	12.513	10.265	246.795
VAR	8.857	10.757	228.606

**Table 11:** Daily comparison of RMSE, SSIM, and centroid distance of case 1.

Metric	Prediction day						
	8	9	10	11	12	13	14
RMSE	**6.913**	7.816	7.491	6.622	7.337	7.719	6.532
SSIM	**0.966**	0.963	0.959	0.961	0.960	0.965	0.939
Cent-Dist in pixels	**0.970**	3.240	2.509	1.360	2.126	1.761	2.809
Cent-Dist in km	**23.331**	77.901	60.333	32.700	51.121	42.334	67.526

**Table 12:** Daily comparison of RMSE, SSIM, and centroid distance of case 2.

Metric	Prediction day						
	8	9	10	11	12	13	14
RMSE	**4.803**	6.484	6.166	5.390	7.190	6.112	6.942
SSIM	0.967	0.960	0.962	0.971	0.959	**0.973**	0.962
Cent-Dist in pixels	**1.833**	4.342	4.047	3.647	5.603	3.085	1.833
Cent-Dist in km	**44.067**	104.385	97.315	87.694	134.723	74.167	44.067

**Table 13:** Daily comparison of RMSE, SSIM, and centroid distance of case 4.

	Prediction day T						
	8	9	10	11	12	13	14
RMSE	7.243	6.949	7.042	7.735	8.240	**6.286**	7.904
SSIM	0.955	0.964	**0.976**	0.971	0.965	0.967	0.947
Cent-Dist in pixels	2.777	3.135	2.749	3.799	3.475	**1.642**	4.182
Cent-Dist in km	66.777	75.379	66.104	91.344	83.544	**39.483**	100.552

**Table 14:** Daily comparison of RMSE, SSIM, and centroid distance of case 6.

	Prediction day T											
	13	14	15	16	17	18	19	20	21	22	23	24
RMSE	**4.16**	4.18	4.77	7.90	5.08	4.35	4.49	5.82	6.18	5.62	4.83	7.16
SSIM	**0.97**	0.96	0.96	0.95	0.96	0.97	0.97	0.96	0.95	0.96	0.96	0.96
Cent-Dist in pixels	**1.5**	1.8	1.5	3.6	2.2	2.3	2.4	3.4	2.1	2.2	2.5	2.4
Cent-Dist in km	**36.7**	45.5	36.9	88.8	53.2	56.1	57.7	82.9	50.8	53.1	61.6	59.3

**Table 15:** Daily comparison of RMSE, SSIM, and centroid distance of case 9.

	Prediction day T											
	13	14	15	16	17	18	19	20	21	22	23	24
RMSE	**4.54**	7.98	15.64	15.11	16.36	16.27	15.36	15.49	14.37	8.39	7.67	7.06
SSIM	**0.97**	0.96	0.93	0.94	0.92	0.93	0.93	0.93	0.94	0.95	0.95	0.96
Cent-Dist in pixels	**3.6**	7.0	13.7	16.4	16.1	15.9	15.7	14.1	10.4	5.7	6.2	5.4
Cent-Dist in km	**87.7**	170.2	329.6	394.5	388.5	383.7	377.9	338.9	250.0	139.2	150.8	130.3

**Table 16:** Daily comparison of RMSE, SSIM, and centroid distance of case 10.

	Prediction day T											
	13	14	15	16	17	18	19	20	21	22	23	24
RMSE	**9.80**	10.55	9.41	10.10	11.57	9.39	10.51	10.45	15.43	19.18	12.82	9.91
SSIM	**0.95**	0.95	0.94	0.94	0.94	0.94	0.94	0.93	0.90	0.90	0.93	0.95
Cent-Dist in pixels	**1.4**	1.6	1.9	3.6	2.9	2.7	3.4	3.8	8.3	15.5	12.2	3.2
Cent-Dist in km	**34.1**	40.5	46.7	88.2	70.3	65.0	82.7	93.4	201.7	373.8	294.7	78.21
